# 

**Published:** 1891-12-12

**Authors:** 


					WlNBORNE'S ^ H
for INFANTS TT JH
ISINGUS
and INYAUDS.
No. 272. Vol.SXI.]^ Saturday,
December 12th, 1891.
'[One Penny.

				

